# Translational issues in precision medicine in neuropathic pain

**DOI:** 10.1080/24740527.2020.1720502

**Published:** 2020-02-05

**Authors:** Anthony H. Dickenson, Ryan Patel

**Affiliations:** Department of Neuroscience, Physiology and Pharmacology, University College London, London, UK

**Keywords:** translational research, neuropathic pain, sensory phenotype, precision medicine, animal models of neuropathy

## Abstract

Neuropathic pain remains poorly treated, with most new drugs falling through the translational gap. The traditional model of bench-to-bedside research has relied on identifying new mechanisms/targets in animal models and then developing clinical applications. Several have advocated bridging the translational gap by beginning with clinical observations and back-translating to animal models for further investigation of mechanisms. There is good evidence that phenotyping of patients through quantitative sensory testing can lead to improved treatment selection and hence improved patient outcomes. This practice has been widely adopted in clinical investigations, but its application in preclinical research is not mainstream. In this review, we retrospectively examine our historical rodent data sets with the aim of reconsidering drug effects on sensory neuronal endpoints, their alignment with clinical observations, and how these might guide future clinical studies.

## RÉSUMÉ

La douleur neuropathique reste mal traitée, alors que la plupart des nouveaux médicaments ne réussissent pas à franchir le fossé translationnel. Le modèle traditionnel de la recherche, du laboratoire au chevet du patient, repose sur l'identification de nouveaux mécanismes ou cibles dans des modèles animaux, suivie du développement d'applications cliniques. Certains préconisent de combler le fossé de la recherche translationnelle en commençant par des observations cliniques et en les transposant ensuite sur des modèles animaux afin d’approfondir l'étude des mécanismes. Il est bien établi que le phénotypage des patients par des tests sensoriels quantitatifs peut conduire à une meilleure sélection des traitements et, par conséquent, à de meilleurs résultats pour les patients. Ces pratiques ont été largement adoptées dans les enquêtes cliniques, mais leur application dans la recherche préclinique n'est pas généralisée. Dans cette revue, nous examinons rétrospectivement nos ensembles de données historiques sur les rongeurs dans le but de reconsidérer les effets des médicaments sur les paramètres neuronaux sensoriels, leur alignement avec les observations cliniques et la manière dont celles-ci pourraient orienter les études cliniques futures.

## Introduction

In many areas of medicine the aim is to move toward personalized or precision treatments so that the therapy matches the cause of the condition. Emphasis has been on defining the genetic cause, but it is rare that a single gene mutation forms the basis for a disorder, and even if this is the case a therapy that manipulates the gene produce is often lacking. In the case of pain, the best characterized genetic bases are mutations in sodium channels with loss and gains of function, depending on the particular changes in Na_v_1.7. A loss-of-function mutation renders the carrier analgesic, thus validating the target.^1^ Blockers of this channel are being developed but are not generally available for human use. In the case of inherited erythromelalgia, a gain-of-function alteration in the channel, the particular mutation in the amino acid sequence confers differential sensitivity of individual patients to the nonspecific sodium channel blockers carbamazepine and mexiletine.^2,3^ This suggests that even with a genetic basis for the pain disorder, drug choice can be complicated. In the case of neuropathic pain, a number of drugs with very different mechanisms of action are approved but the numbers needed to treat is high, circa six, indicating that only a minority of patients will gain relief with a particular drug.^4^ This is perhaps not surprising, because although peripheral neuropathic pain originates at the site of the damaged nerves, there are considerable pharmacological changes at this level as well as many and varied changes in the spinal cord, brain, and descending controls. Because each drug has a defined action, one could presume that in order to treat the pain adequately, the drug would have to target the predominant mechanism active in that patient.

**Figure 1.**
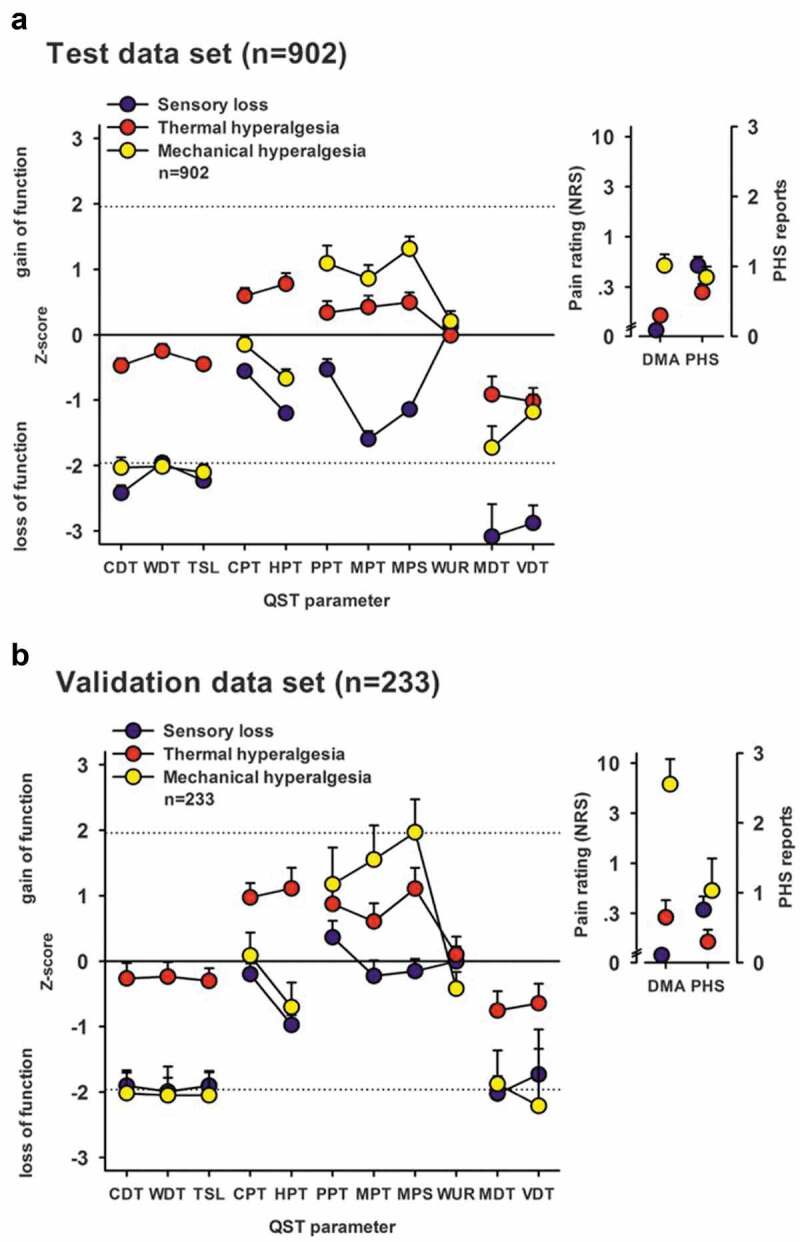
Sensory profiles of the three-cluster solution for test and replication data sets. Sensory profiles of the three clusters are presented as mean *z* scores ± 95% confidence interval for (A) the test data set (*n* = 902) and (B) the validation data set (*n* = 233). Note that *z* transformation eliminates differences due to test site, sex, and age. Positive *z* scores indicate positive sensory signs (hyperalgesia), whereas negative *z* values indicate negative sensory signs (hypoesthesia and hypoalgesia). Dashed lines: 95% confidence interval for healthy subjects (−1.96 < *z* < + 1.96). Note that if the mean of a cluster is within the shaded area, this does not imply that it does not differ from a healthy cohort. Values are significantly different from those of healthy subjects if the 95% confidence interval does not cross the zero line. Insets show numeric pain ratings for dynamic mechanical allodynia on a logarithmic scale (0–100) and frequency of paradoxical heat sensation (0–3). Blue symbols: Cluster 1 “sensory loss” (42% in A and 53% in B). Red symbols: Cluster 2 “thermal hyperalgesia” (33% in A and B). Yellow symbols: Cluster 3 “mechanical hyperalgesia” (24% in A and 14% in B). CDT = cold detection threshold; WDT = warm detection threshold; TSL = thermal sensory limen; CPT = cold pain threshold; HPT = heat pain threshold; PPT = pressure pain threshold; MPT = mechanical pain threshold; MPS = mechanical pain sensitivity; WUR = windup ratio; MDT = mechanical detection threshold; VDT = vibration detection threshold; NRS = Numerical Rating Scale; DMA = dynamic mechanical allodynia; PHS = paradoxical heat sensation. Reproduced with permission from Baron et al.^5^ (under a Creative Commons license)

In the rest of this review, we will discuss a number of other drugs used for neuropathic pain and their profiles in preclinical models. There is a key issue here regarding translation. If a drug has effects on particular responses or modalities, then many drugs may have failed in clinical trials where patients were simply included based on a particular etiology, whether postherpetic, diabetic, HIV, or chemotherapy-induced neuropathy. It has been clearly shown that independent of the etiology, patients with neuropathic pain fall into three subgroups, based on their sensory profiles determined through quantitative sensory testing (QST; Figure 1).^5^ Similar separable subgroups of patients have been identified among patients with fibromyalgia and postsurgical pain, suggesting that the heterogeneity crosses pain syndromes. Failing to take this into account means that the patients in the trials were very likely to be heterogeneous in terms of mechanisms and thus drugs that were effective in some patients would not be observed and the trial and drug would fail. Consequently, the idea of a mechanism-based rationale for bench-to-bedside translation when screening novel drugs is a crucial point because it will guide the trial design.

To align sensory profiling in clinical and preclinical studies, suitable measures of sensory processing are required. At present, probing of somatosensory function in rodents is largely accomplished by determining paw withdrawal thresholds or by performing neurophysiology. The “all-or-nothing” nature of a withdrawal response poses certain limitations. Below threshold there is no quantitative measure of any sensory processing, a response is only measured when threshold is breached, and above-threshold responses cannot be readily quantified because the test is effectively terminated at the first point of withdrawal. Secondly, these behaviors are largely governed by spinobulbal processing mechanisms with limited cortical involvement. In recent years, novel endpoints have been developed, such as classical or operant-based conditioned place aversion to sensory stimuli,^6,7^ and these tests provide additional insight into the affective/aversive dimension of pain. These measures of cognitive function may be more translatable than reflexive endpoints. Neurophysiology can overcome some of these limitations by providing a quantitative and objective measure of sensory neuronal transmission. Microneurography, for example, can be performed in both rodents and humans,^8^ and numerous studies have established the relationship of second- and third-order neuronal firing with perceptual outcomes.^9–12^ We retrospectively examine the effects of drugs on this latter neuronal endpoint in rats from our historical data sets (summarized in Table 1). The chosen endpoints encompass several of the modalities applied during QST, such as dynamic brush, punctate mechanical, and heat and cold, as well as measures such windup. Drug effects against innocuous and noxious intensities of stimulation are also considered, and these may better relate to the range of intensities used during QST. Lastly, conditioned pain modulation (CPM), analogous to diffuse noxious inhibitory controls (DNICs) in animal models, is often performed alongside QST and is also discussed.

**Table 1.** Effects of drugs on spinothalamic wide dynamic range neuronal responses in spinal nerve ligated rats

			Punctate mechanical		Heat		Cold				
Receptor	Drug	Brush	Innocuous	Noxious	Innocuous	Noxious	Innocuous	Noxious	Windup	Spontaneous	DNIC
VGSCs	Oxcarbazepine (s.c)^14^	↓	↓↓	↓↓	–	–	↓	↓↓	–	↓↓	
VGSCs	Licarbazepine (i.pl)^14^	↓	↓↓	↓↓	–	–	↓	↓↓	–	↓↓	
5-HT_2A_	Ketanserin (i.th)^58^	–	–	↓	–	↓	↓↓	↓↓		–	
NET	Reboxetine (i.th)^56,61^	–	↓	↓	–	–	–	↓		–	↑↑
TRPM8	M8-an (s.c)^62^	–	–	–	–	–	↓↓	↓↓	–		
α_2_δ-1	Pregabalin (s.c)^31,41^	↓↓	↓↓	↓↓	–	↓	–	–	–	–	
5-HT_3_	Ondansetron (i.th)^56,58,63^	–	↓↓	↓↓	–	↓↓	–	–	–	–	↑↑
Ca_v_2.1/2/3	TROX-1 (i.th & s.c)^50^	–	↓↓	↓↓	–	–	–	–	–		
OR	Morphine (s.c)^64^	–	↓↓	↓↓	↓↓	↓↓		↓↓		↓↓	
α_2_	Clonidine (i.th)^61^	↓↓	↓↓	↓↓	↓↓	↓↓	↓↓	↓↓		↓↓	
μOR/NET	Tapentadol (s.c)^56,65^	↓↓	↓↓	↓↓	↓	↓↓			↓↓		↑↑
Ca_v_2.1/3	Tx3-3 (i.th)^49^	↓	↓↓	↓↓	↓	↓↓			↓		
A_3_	MRS5698 (s.c)^66^	↓	↓↓	↓↓	↓↓	↓↓			↓↓		
VGSCs	Lacosamide (i.th & s.c)^15^	↓	–	↓↓	↓	↓↓			↓↓		
NMDA	Ketamine (s.c)^67,68^		↓↓	↓↓		↓↓			↓↓	–	

Examining drug actions on evoked sensory neuronal endpoints reveals three broad groups characterized by predominant inhibitory effects on (1) mechanically and cold-evoked responses, (2) mechanically evoked responses, and (3) all modalities.

↓ = moderately inhibited; ↓↓ = inhibited; ↑↑ = enhanced; – = no/minimal effect; (blank) = not tested; VGSCs = voltage-gated sodium channels; s.c = subcutaneous; i.pl = intraplantar; i.th = intrathecal; NET = norepinephrine transporter; TRPM8 = Transient Receptor Potential Melastatin 8; OR = opioid receptor; A = adenosine.

## Irritable Nociceptors

To decipher a mechanism in a particular patient at any one time is next to impossible at present. An approach that would overcome this problem would be to reverse the process and work from the premise that the sensory signs and symptoms of the patient must reflect the underlying mechanisms at play and that drugs have a defined mode of action. Thus, attempting to align the sensory phenotype of the patient with a particular class of drug could be very fruitful. The overall aim would be to define patient subgroups and relate them to the efficacy of a particular drug. This has been achieved with the sodium channel blocker oxcarbazepine. In a randomized controlled trial with patients with neuropathic pain, the drug did not separate from placebo.^13^ However, when patients with a so-called irritable nociceptor who were suffering from evoked pain hypersensitivity were split off, the drug was effective. Thus, abnormal impulses in still-connected nerves in these patients appear to be driving the pain and thus blocking this activity through sodium channel modulation alleviates the pain.

Based on this study, we recorded activity in thalamic neurons in a model of neuropathy and studied the effects of the drug and the effects of lidocaine, another sodium channel blocker.^14^ Because the clinical study had used systemic administration, we also aimed to localize the site of action of these ion channel blockers. We studied both ongoing and evoked responses of the neurons in the pathways behind sensory components of pain and spontaneous activity in the thalamus. A marked reduction in spontaneous activity was seen after spinal lidocaine, with no effect being seen in sham rats. This measure was partly driven by ongoing peripheral activity because intraplantar lidocaine also reduced this ongoing thalamic neuronal firing. Systemic oxcarbazepine in neuropathic animals markedly inhibited evoked responses, namely, punctate mechanical, dynamic brush- and cold-evoked neuronal responses in the thalamus and dorsal horn, but did not show a marked effect on heat-evoked firing yet inhibited spontaneous activity in the thalamus. Intraplantar injection of the active metabolite licarbazepine replicated the effects of systemic oxcarbazepine, supporting a peripheral locus of action.

Thus, overall, ongoing activity in primary afferent fibers drives spontaneous thalamic firing after spinal nerve injury and oxcarbazepine exhibits modality-selective inhibitory effects on sensory neuronal processing through a peripheral mechanism. These inhibitory effects of both lidocaine and oxcarbazepine suggest that this rat model of neuropathy, involving a partial ligation of spinal nerves, resembles the irritable nociceptor patient subgroup. Baron and colleagues proposed that these patients are represented within the thermal cluster and that peripheral sensitization is the predominant pathophysiological mechanism.^5^ Spinal nerve injury in rats produces a profile of sensory gain in a manner that resembles the thermal and mechanical phenotypes in patients but has little likeness to the sensory loss group.^14^

Like oxcarbazepine, lacosamide is a nonselective sodium channel blocker and also reduced evoked spinal neuronal responses in an experimental rat model.^15^ Based on a meta-analysis of clinical trial data, lacosamide has limited to no benefit compared to placebo in an overall patient population^16^; however, as with oxcarbazepine, this may stem from a lack of patient stratification rather than lack of efficacy as such. A recently registered trial will attempt to address this by investigating whether a similar drug-sensory phenotype interaction exists.^17^ A multimodal genetic, electrophysiological, and sensory profiling approach has already showed promise for personalized treatment selection; several recent studies support that patients with Na_v_1.7 variant-driven small fiber neuropathies can benefit from lacosamide treatment.^18–20^

## Calcium Channel Modulators

Despite accumulating vast global annual sales, the α_2_δ-1/2 ligands pregabalin and gabapentin are known to be ineffective for the majority of patients with neuropathic pain.^4^ Their effects have been comprehensively characterized in rodent injury models, and both drugs attenuate ongoing pain and evoked hypersensitivity through central mechanisms, particularly where central sensitization is present.^21–23^ In human surrogate models of central sensitization, the gabapentinoid drugs are more effective at reducing areas of secondary pinprick hyperalgesia rather than altering mechanical pain thresholds.^24–27^ Clearly, rodent models of nerve injury recapitulate underlying mechanisms that are shared with a proportion of patients who are sensitive to gabapentinoid treatment (reviewed in detail in Patel and Dickenson^28^).

In a case study of spinal cord injury, a patient with ongoing pain presented with contrasting left and right QST profiles at the same thoracic level. Intriguingly, these pains exhibited different sensitivity to pregabalin treatment. Where there was fiber loss and sensory deficits, ongoing pain was refractory to pregabalin treatment but pain relief was achieved in the region where sensory function was preserved.^29^ Although only a single example, it illustrates how careful dissection of mechanisms can distinguish different pains and perhaps aid treatment selection. It is conceivable that in clinical trials, patients with mixed pains may report insufficient pain relief due to targeting of a single neurobiological mechanism. When tested against QST endpoints in a small group of patients with peripheral neuropathy, chronic gabapentin treatment reduced brush allodynia and raised cold pain thresholds where these sensory disturbances were present but had no effect on heat detection or pain thresholds in all patients tested or any effect on punctate mechanical stimuli in patients with mechanical hyperalgesia.^30^ Although low in numbers, this study displays some concordance with the effects of acute pregabalin on sensory neuronal processing in rats with peripheral nerve injury because brush-evoked neuronal responses were inhibited and heat-evoked responses were only weakly affected, but contrasting effects were seen with respect to cold and mechanical stimuli.^31^ In the rat models at least, the gabapentinoids are particularly effective at inhibiting high-intensity mechanically evoked neuronal responses. On a larger scale, post hoc analysis of clinical trial data revealed that pregabalin had no benefit in patients with HIV neuropathy but provided pain relief in a patient subgroup characterized by severe pinprick hyperalgesia.^32^ A more recent study of patients with chemotherapy-induced neuropathy found that mechanical pain thresholds were not predictive of response to pregabalin, though the number of patients exhibiting mechanical hypersensitivity was low,^33^ and the former study noted that only those patients with the most severe pinprick hyperalgesia benefited, whereas those with mild/moderate hyperalgesia did not. Baron and colleagues proposed that these patients are represented by the mechanical cluster (Figure 1) and that central sensitization is the predominant pathophysiological mechanism.^5^ Consistent with the features of this sensory phenotype, post hoc analysis of a separate pregabalin trial concluded that analgesia corresponded with preserved large fiber function and poorer outcomes were observed with loss of fibers.^34^

Windup and temporal summation of pain and conditioned pain modulation are two measures that could merit further investigation. Windup is commonly interpreted as a readout for sensitization state. Electrically evoked windup produces hyperexcitability of spinal neurons that induces transient features that are shared with central sensitization, such as enlarged receptive field sizes and windup at lower frequencies.^35^ Moreover, windup and the human counterpart test temporal summation share similar pharmacological properties—for example, abolished by N-methyl-d-aspartate (NMDA) block^36,37^—and patients with neuropathy with enhanced temporal summation are more likely to benefit from ketamine treatment.^38^ In pain-free volunteers, the ability of gabapentin and pregabalin to inhibit temporal summation of pain is dependent on the sensitization state.^39,40^ Data from animal models are rather mixed; neither gabapentin nor pregabalin inhibits windup in uninjured rats, but whether windup is inhibited in neuropathy seems to depend on the precise nature of the nerve injury.^21,41–43^ Supraspinal analgesic mechanisms of the gabapentinoid drugs include activating descending inhibitory pathways,^23,44^ though at present there appears to be no report of whether they improve CPM in patients with neuropathy. In a nonneuropathic condition such as pancreatitis, pregabalin had no effect on low CPM responses.^45^ In rats at least, pregabalin does not restore deficient DNICs in a model of knee degeneration where disrupted descending modulation forms part of the etiology of persistent joint pain.^46^

Gabapentin was in use as an anticonvulsant but found new purpose as an analgesic following a clinical observation. One of the rare success stories for bench-to-bedside translation was the discovery that intrathecal ω-conotoxin was antinociceptive, which led to the development of ziconotide, a synthetic peptide blocker of the voltage-gated calcium channel Ca_v_2.2.^47^ The poor blood–brain barrier penetration necessitates delivery via the intrathecal route and, due to this impracticality, it is most commonly used in cases refractory to treatment by other drugs. Ziconotide can provide relief for many of these patients, validating targeting of calcium channels for treatment of neuropathic pain.^48^ Like the conotoxins, the spider toxin Tx3-3 has broad inhibitory effects on spinal neuronal responses consistent with its lack of selectivity over channel subtypes and activation state.^49^ The development of small molecule activation state-dependent blockers was intended to circumvent the neurological side effects that can be associated with peptide blockers, the hypothesis being that targeting of calcium channels in hyperexcitable primary afferents would block aberrant nociceptive signaling while permitting normal sensory transmission. Several of the developed compounds (CNV219794, Ca_v_2.2; ABT-639, Ca_v_3.2; Z160, Ca_v_2.2) displayed good tolerability within phase I trials but failed to demonstrate efficacy in phase II (NCTs: 1655849, 01345045, 01848730, 01893125). In line with the predicted mechanism of action, the activation state-dependent but non-subtype-selective Ca_v_2 blocker TROX-1 has no effect on normal sensory transmission but exhibits modality-selective effects in neuropathic rats, only inhibiting responses evoked by punctate mechanical stimuli.^50^ All of the failed trials selected patients on the basis of disease etiology and did not distinguish between those with sensory gain and sensory loss. Drawing parallels with the oxcarbazepine trial, these drugs may be particularly effective where pain is driven by peripheral hyperexcitability and evoked pain is the primary sensory disturbance.

## Descending Control

Over 40 years ago, DNICs were described whereby a distant noxious stimulus would inhibit the responses of spinal wide dynamic range neurons through descending controls.^51^ Subsequently, it has been shown that these neurons are part of the spinothalamic tract, code the intensity and spatial summation of a noxious thermal stimulus in an overlapping way with the human pain ratings to the same stimulus,^12^ and exhibit both peripheral and central sensitization after ultraviolet B radiation and heat, corresponding with the human psychophysical correlates of the same model.^11^ This correspondence between neuronal coding (even under general anesthesia) and human sensory reports is likely the basis for why CPM, the human counterpart of DNICs, can be readily established in volunteers and patients. In many patients with persistent pain there is a reduction in CPM, and a low CPM at the time of surgery is a risk factor for persistent pain.^52,53^ Have tested CPM in patients and studied the interaction between pain control and this descending inhibitory system. Duloxetine (a serotonin–noradrenaline reuptake inhibitor) and tapentadol (a dual μ-opioid receptor agonist/noradrenaline reuptake inhibitor) were tested in patients with diabetic neuropathy. Duloxetine efficacy was predicted by CPM in that low or lost CPM correlated with drug effectiveness, whereas tapentadol restored CPM as its analgesic effect appeared.^54,55^ Both studies are explained by the subsequent preclinical finding that DNICs were fully mediated by noradrenaline acting at the α_2_-adrenoceptor, that it was lost in models of nerve injury and could be restored by both tapentadol and reboxetine, the noradrenaline reuptake inhibitor.^56^ Very similar findings were seen in a model of osteoarthritis, in agreement with human data where CPM is lost in many pain states.^57^

DNICs are noradrenergic inhibitors but are balanced by descending facilitations mediated by 5-Hydroxytryptamine (HT) largely through the 2A and 3 receptors. A large number of preclinical studies have shown that a loss of descending inhibition, normally protective, is accompanied by a gain of facilitation as pain becomes persistent, and recent human imaging studies in very different pain states, namely, fibromyalgia and severe osteoarthritis, report similar findings. These serotonergic enhancements of pain can be blocked by antagonists such as ondansetron (5-HT_3_R) and ketanserin (5-HT_2A_R).^58^ When given spinally, the former drug restores DNICs and has an enhanced action on sensory coding of spinal and thalamic neurons in models of nerve injury.^56,58^ Two human studies have used the drug in patients with neuropathy, and one was positive when ongoing pain was the outcome measure, whereas the other, looking at dynamic allodynia and ongoing pain, was negative.^59,60^ However, these discrepant results are explicable on the basis that the drug has differential effects on neuronal coding with modulation of punctate mechanical and heat stimuli but no effect on brush or ongoing activity. This emphasizes the point that clinical studies may “fail” if the wrong endpoints are picked.

## Summary

It is becoming increasingly clear that pharmacological agents for the treatment of neuropathic pain have differential effects on different modalities/ongoing activity and that this can be readily examined in preclinical models measuring the activity of spinal and brain neurons. Therefore, designing a trial based on etiology seems highly likely to fail to identify those patients who will respond to treatment, and indeed many have failed in recent years. Simple reflexive withdrawal responses in preclinical development of drugs are not able to dissect out the nuances of drug actions and thus will not be helpful in providing a basis for mechanism-based treatments. Behavioral assays requiring cognitive processing offer greater insight but are not suited to measuring windup/temporal summation or DNICs where neurophysiological measures are advantageous. From this account, we would recommend a reconsideration of some of the pharmacological agents that have failed in trials based simply on etiology. Most of the preclinical and clinical data are based on neuropathic pain, but the same principles may apply to other important conditions such as osteoarthritis and fibromyalgia.

## References

[1] Cox JJ, Reimann F, Nicholas AK, Thornton G, Roberts E, Springell K, Karbani G, Jafri H, Mannan J, Raashid Y, et al. An SCN9A channelopathy causes congenital inability to experience pain. Nature. 2006;444(7121):894–98. doi:10.1038/nature05413. 17167479 PMC7212082

[2] Choi J-S, Zhang L, Dib-Hajj SD, Han C, Tyrrell L, Lin Z, Wang X, Yang Y, Waxman SG. Mexiletine-responsive erythromelalgia due to a new Na(v)1.7 mutation showing use-dependent current fall-off. Exp Neurol. 2009;216(2):383–89. doi:10.1016/j.expneurol.2008.12.012. 19162012

[3] Fischer TZ, Gilmore ES, Estacion M, Eastman E, Taylor S, Melanson M, Dib-Hajj SD, Waxman SG. A novel Nav1.7 mutation producing carbamazepine-responsive erythromelalgia. Ann Neurol. 2009;65(6):733–41. doi:10.1002/ana.v65:6. 19557861 PMC4103031

[4] Finnerup NB, Attal N, Haroutounian S, McNicol E, Baron R, Dworkin RH, Gilron I, Haanpaa M, Hansson P, Jensen TS, et al. Pharmacotherapy for neuropathic pain in adults: a systematic review and meta-analysis. Lancet Neurol. 2015;14(2):162–73. doi:10.1016/S1474-4422(14)70251-0. 25575710 PMC4493167

[5] Baron R, Maier C, Attal N, Binder A, Bouhassira D, Cruccu G, Finnerup NB, Haanpaa M, Hansson P, Hullemann P, et al. Peripheral neuropathic pain: a mechanism-related organizing principle based on sensory profiles. Pain. 2017;158(2):261–72. doi:10.1097/j.pain.0000000000000753. 27893485 PMC5266425

[6] Cheng L, Duan B, Huang T, Zhang Y, Chen Y, Britz O, Garcia-Campmany L, Ren X, Vong L, Lowell BB, et al. Identification of spinal circuits involved in touch-evoked dynamic mechanical pain. Nat Neurosci. 2017;20(6):804–14. doi:10.1038/nn.4549. 28436981 PMC5470641

[7] Hayashida K-I, Eisenach JC, Kawatani M, Martin TJ. Peripheral nerve injury in rats induces alternations in choice behavior associated with food reinforcement. J Physiol Sci. 2019;69(5):769–77. doi:10.1007/s12576-019-00693-6. 31267368 PMC10717269

[8] Ackerley R, Watkins RH. Microneurography as a tool to study the function of individual C-fiber afferents in humans: responses from nociceptors, thermoreceptors, and mechanoreceptors. J Neurophysiol. 2018;120(6):2834–46. doi:10.1152/jn.00109.2018. 30256737

[9] Coghill RC, Mayer DJ, Price DD. Wide dynamic range but not nociceptive-specific neurons encode multidimensional features of prolonged repetitive heat pain. J Neurophysiol. 1993;69(3):703–16. doi:10.1152/jn.1993.69.3.703. 8385190

[10] Dubner R, Kenshalo DR Jr., Maixner W, Bushnell MC, Oliveras JL. The correlation of monkey medullary dorsal horn neuronal activity and the perceived intensity of noxious heat stimuli. J Neurophysiol. 1989;62(2):450–57. doi:10.1152/jn.1989.62.2.450. 2769341

[11] O’Neill J, Sikandar S, McMahon SB, Dickenson AH. Human psychophysics and rodent spinal neurones exhibit peripheral and central mechanisms of inflammatory pain in the UVB and UVB heat rekindling models. J Physiol. 2015;593(17):4029–42. doi:10.1113/JP270294. 26047369 PMC4575584

[12] Sikandar S, Ronga I, Iannetti GD, Dickenson AH. Neural coding of nociceptive stimuli-from rat spinal neurones to human perception. Pain. 2013;154(8):1263–73. doi:10.1016/j.pain.2013.03.041. 23719576

[13] Demant DT, Lund K, Vollert J, Maier C, Segerdahl M, Finnerup NB, Jensen TS, Sindrup SH. The effect of oxcarbazepine in peripheral neuropathic pain depends on pain phenotype: a randomised, double-blind, placebo-controlled phenotype-stratified study. Pain. 2014;155(11):2263–73. doi:10.1016/j.pain.2014.08.014. 25139589

[14] Patel R, Kucharczyk M, Montagut-Bordas C, Lockwood S, Dickenson AH. Neuropathy following spinal nerve injury shares features with the irritable nociceptor phenotype: A back-translational study of oxcarbazepine. Eur J Pain. 2019;23(1):183–97. doi:10.1002/ejp.2019.23.issue-1. 30091265 PMC6396087

[15] Bee LA, Dickenson AH. Effects of lacosamide, a novel sodium channel modulator, on dorsal horn neuronal responses in a rat model of neuropathy. Neuropharmacology. 2009;57(4):472–79. doi:10.1016/j.neuropharm.2009.06.021. 19573541

[16] Hearn L, Derry S, Moore RA. Lacosamide for neuropathic pain and fibromyalgia in adults. Cochrane Database Syst Rev. 2012;40(2):CD009318. 22336864 10.1002/14651858.CD009318.pub2 PMC8406928

[17] Carmland ME, Kreutzfeldt M, Holbech JV, Andersen NT, Jensen TS, Bach FW, Sindrup SH, Finnerup NB. Effect of lacosamide in peripheral neuropathic pain: study protocol for a randomized, placebo-controlled, phenotype-stratified trial. Trials. 2019;20(1):588. doi:10.1186/s13063-019-3695-7. 31604475 PMC6788106

[18] Blesneac I, Themistocleous AC, Fratter C, Conrad LJ, Ramirez JD, Cox JJ, Tesfaye S, Shillo PR, Rice ASC, Tucker SJ, et al. Rare NaV1.7 variants associated with painful diabetic peripheral neuropathy. Pain. 2018;159(3):469–80. doi:10.1097/j.pain.0000000000001116. 29176367 PMC5828379

[19] de Greef BTA, Hoeijmakers JGJ, Geerts M, Oakes M, Church TJE, Waxman SG, Dib-Hajj SD, Faber CG, Merkies ISJ. Lacosamide in patients with Nav1.7 mutations-related small fibre neuropathy: a randomized controlled trial. Brain. 2019;142(2):263–75. doi:10.1093/brain/awy329. 30649227

[20] Namer B, Schmidt D, Eberhardt E, Maroni M, Dorfmeister E, Kleggetveit IP, Kaluza L, Meents J, Gerlach A, Lin Z, et al. Pain relief in a neuropathy patient by lacosamide: proof of principle of clinical translation from patient-specific iPS cell-derived nociceptors. EBioMedicine. 2019;39:401–08. doi:10.1016/j.ebiom.2018.11.042. 30503201 PMC6354557

[21] Bannister K, Qu C, Navratilova E, Oyarzo J, Xie JY, King T, Dickenson AH, Porreca F. Multiple sites and actions of gabapentin-induced relief of ongoing experimental neuropathic pain. Pain. 2017;158(12):2386–95. doi:10.1097/j.pain.0000000000001040. 28832395 PMC5681862

[22] Hunter JC, Gogas KR, Hedley LR, Jacobson LO, Kassotakis L, Thompson J, Fontana DJ. The effect of novel anti-epileptic drugs in rat experimental models of acute and chronic pain. Eur J Pharmacol. 1997;324(2–3):153–60. doi:10.1016/S0014-2999(97)00070-8. 9145766

[23] Juarez-Salinas DL, Braz JM, Hamel KA, Basbaum AI. Pain relief by supraspinal gabapentin requires descending noradrenergic inhibitory controls. Pain Rep. 2018;3(4):e659. doi:10.1097/PR9.0000000000000659. 30123855 PMC6085145

[24] Chizh BA, Gohring M, Troster A, Quartey GK, Schmelz M, Koppert W. Effects of oral pregabalin and aprepitant on pain and central sensitization in the electrical hyperalgesia model in human volunteers. Br J Anaesth. 2007;98(2):246–54. doi:10.1093/bja/ael344. 17251214

[25] Dirks J, Petersen KL, Rowbotham MC, Dahl JB. Gabapentin suppresses cutaneous hyperalgesia following heat-capsaicin sensitization. Anesthesiology. 2002;97(1):102–07. doi:10.1097/00000542-200207000-00015. 12131110

[26] Segerdahl M. Multiple dose gabapentin attenuates cutaneous pain and central sensitisation but not muscle pain in healthy volunteers. Pain. 2006;125(1–2):158–64. doi:10.1016/j.pain.2006.05.008. 16781073

[27] Werner MU, Perkins FM, Holte K, Pedersen JL, Kehlet H. Effects of gabapentin in acute inflammatory pain in humans. Reg Anesth Pain Med. 2001;26(4):322–28. doi:10.1097/00115550-200107000-00008. 11464350

[28] Patel R, Dickenson AH. Mechanisms of the gabapentinoids and α2δ-1 calcium channel subunit in neuropathic pain. Pharmacol Res Perspect. 2016;4(2):e00205. doi:10.1002/prp2.205. 27069626 PMC4804325

[29] Westermann A, Krumova EK, Pennekamp W, Horch C, Baron R, Maier C. Different underlying pain mechanisms despite identical pain characteristics: a case report of a patient with spinal cord injury. Pain. 2012;153(7):1537–40. doi:10.1016/j.pain.2012.02.031. 22444186

[30] Attal N, Brasseur L, Parker F, Chauvin M, Bouhassira D. Effects of gabapentin on the different components of peripheral and central neuropathic pain syndromes: a pilot study. Eur Neurol. 1998;40(4):191–200. doi:10.1159/000007979. 9813401

[31] Patel R, Dickenson AH. Neuronal hyperexcitability in the ventral posterior thalamus of neuropathic rats: modality selective effects of pregabalin. J Neurophysiol. 2016;116(1):159–70. doi:10.1152/jn.00237.2016. 27098028 PMC4961752

[32] Simpson DM, Schifitto G, Clifford DB, Murphy TK, Durso-De Cruz E, Glue P, Whalen E, Emir B, Scott GN, Freeman R, et al. Pregabalin for painful HIV neuropathy: a randomized, double-blind, placebo-controlled trial. Neurology. 2010;74(5):413–20. doi:10.1212/WNL.0b013e3181ccc6ef. 20124207 PMC2816006

[33] Hincker A, Frey K, Rao L, Wagner-Johnston N, Ben Abdallah A, Tan B, Amin M, Wildes T, Shah R, Karlsson P, et al. Somatosensory predictors of response to pregabalin in painful chemotherapy-induced peripheral neuropathy: a randomized, placebo-controlled, crossover study. Pain. 2019;160(8):1835–46. doi:10.1097/j.pain.0000000000001577. 31335651 PMC6687437

[34] Holbech JV, Bach FW, Finnerup NB, Jensen TS, Sindrup SH. Pain phenotype as a predictor for drug response in painful polyneuropathy-a retrospective analysis of data from controlled clinical trials. Pain. 2016;157(6):1305–13. doi:10.1097/j.pain.0000000000000563. 27007067

[35] Herrero JF, Laird JM, Lopez-Garcia JA. Wind-up of spinal cord neurones and pain sensation: much ado about something? Prog Neurobiol. 2000;61(2):169–203. doi:10.1016/S0301-0082(99)00051-9. 10704997

[36] Dickenson AH, Sullivan AF. Evidence for a role of the NMDA receptor in the frequency dependent potentiation of deep rat dorsal horn nociceptive neurones following C fibre stimulation. Neuropharmacology. 1987;26(8):1235–38. doi:10.1016/0028-3908(87)90275-9. 2821443

[37] Price DD, Mao J, Frenk H, Mayer DJ. The N-methyl-D-aspartate receptor antagonist dextromethorphan selectively reduces temporal summation of second pain in man. Pain. 1994;59(2):165–74. doi:10.1016/0304-3959(94)90069-8. 7892014

[38] Bosma RL, Cheng JC, Rogachov A, Kim JA, Hemington KS, Osborne NR, Venkat Raghavan L, Bhatia A, Davis KD. Brain dynamics and temporal summation of pain predicts neuropathic pain relief from ketamine infusion. Anesthesiology. 2018;129(5):1015–24. doi:10.1097/ALN.0000000000002417. 30199420

[39] Arendt-Nielsen L, Frokjaer JB, Staahl C, Graven-Nielsen T, Huggins JP, Smart TS, Drewes AM. Effects of gabapentin on experimental somatic pain and temporal summation. Reg Anesth Pain Med. 2007;32(5):382–88. doi:10.1097/00115550-200709000-00004. 17961835

[40] Arendt-Nielsen L, Mansikka H, Staahl C, Rees H, Tan K, Smart TS, Monhemius R, Suzuki R, Drewes AM. A translational study of the effects of ketamine and pregabalin on temporal summation of experimental pain. Reg Anesth Pain Med. 2011;36(6):585–91. doi:10.1097/AAP.0b013e31822b0db0. 21941220

[41] Bee LA, Dickenson AH. Descending facilitation from the brainstem determines behavioural and neuronal hypersensitivity following nerve injury and efficacy of pregabalin. Pain. 2008;140(1):209–23. doi:10.1016/j.pain.2008.08.008. 18809257

[42] Curros-Criado MM, Herrero JF. The antinociceptive effect of systemic gabapentin is related to the type of sensitization-induced hyperalgesia. J Neuroinflammation. 2007;4:15. doi:10.1186/1742-2094-4-15. 17550605 PMC1892010

[43] Ding L, Cai J, Guo XY, Meng XL, Xing GG. The antiallodynic action of pregabalin may depend on the suppression of spinal neuronal hyperexcitability in rats with spared nerve injury. Pain Res Manage. 2014;19(4):205–11. doi:10.1155/2014/623830. PMC4158936 24851240

[44] Tanabe M, Takasu K, Kasuya N, Shimizu S, Honda M, Ono H. Role of descending noradrenergic system and spinal alpha2-adrenergic receptors in the effects of gabapentin on thermal and mechanical nociception after partial nerve injury in the mouse. Br J Pharmacol. 2005;144(5):703–14. doi:10.1038/sj.bjp.0706109. 15678083 PMC1576051

[45] Bouwense SA, Olesen SS, Drewes AM, Poley JW, van Goor H, Wilder-Smith OH. Effects of pregabalin on central sensitization in patients with chronic pancreatitis in a randomized, controlled trial. PLoS One. 2012;7(8):e42096. doi:10.1371/journal.pone.0042096. 22879908 PMC3412837

[46] Lockwood SM, Dickenson AH. A combination pharmacotherapy of tapentadol and pregabalin to tackle centrally driven osteoarthritis pain. Eur J Pain. 2019;23(6):1185–95. doi:10.1002/ejp.2019.23.issue-6. 30821870 PMC6618140

[47] Chaplan SR, Pogrel JW, Yaksh TL. Role of voltage-dependent calcium channel subtypes in experimental tactile allodynia. J Pharmacol Exp Ther. 1994;269:1117–23. 8014856

[48] Sanford M. Intrathecal ziconotide: a review of its use in patients with chronic pain refractory to other systemic or intrathecal analgesics. CNS Drugs. 2013;27(11):989–1002. doi:10.1007/s40263-013-0107-5. 23999971

[49] Dalmolin GD, Bannister K, Goncalves L, Sikandar S, Patel R, Cordeiro MDN, Gomez MV, Ferreira J, Dickenson AH. Effect of the spider toxin Tx3-3 on spinal processing of sensory information in naive and neuropathic rats: an in vivo electrophysiological study. Pain Rep. 2017;2(4):e610. doi:10.1097/PR9.0000000000000610. 29392225 PMC5741365

[50] Patel R, Rutten K, Valdor M, Schiene K, Wigge S, Schunk S, Damann N, Christoph T, Dickenson AH. Electrophysiological characterization of activation state-dependent Ca(v)2 channel antagonist TROX-1 in spinal nerve injured rats. Neuroscience. 2015;297:47–57. doi:10.1016/j.neuroscience.2015.03.057. 25839150 PMC4436437

[51] Le Bars D, Dickenson AH, Besson JM. Diffuse noxious inhibitory controls (DNIC). I. Effects on dorsal horn convergent neurones in the rat. Pain. 1979;6(3):283–304. doi:10.1016/0304-3959(79)90049-6. 460935

[52] Wilder-Smith OH, Schreyer T, Scheffer GJ, Arendt-Nielsen L. Patients with chronic pain after abdominal surgery show less preoperative endogenous pain inhibition and more postoperative hyperalgesia: a pilot study. J Pain Palliat Care Pharmacother. 2010;24(2):119–28. doi:10.3109/15360281003706069. 20504133

[53] Yarnitsky D, Crispel Y, Eisenberg E, Granovsky Y, Ben-Nun A, Sprecher E, Best LA, Granot M. Prediction of chronic post-operative pain: pre-operative DNIC testing identifies patients at risk. Pain. 2008;138(1):22–28. doi:10.1016/j.pain.2007.10.033. 18079062

[54] Niesters M, Proto PL, Aarts L, Sarton EY, Drewes AM, Dahan A. Tapentadol potentiates descending pain inhibition in chronic pain patients with diabetic polyneuropathy. Br J Anaesth. 2014;113(1):148–56. doi:10.1093/bja/aeu056. 24713310

[55] Yarnitsky D, Granot M, Nahman-Averbuch H, Khamaisi M, Granovsky Y. Conditioned pain modulation predicts duloxetine efficacy in painful diabetic neuropathy. Pain. 2012;153(6):1193–98. doi:10.1016/j.pain.2012.02.021. 22480803

[56] Bannister K, Patel R, Goncalves L, Townson L, Dickenson AH. Diffuse noxious inhibitory controls and nerve injury: restoring an imbalance between descending monoamine inhibitions and facilitations. Pain. 2015;156(9):1803–11. doi:10.1097/j.pain.0000000000000240. 26010460

[57] Lockwood S, Dickenson AH. What goes up must come down: insights from studies on descending controls acting on spinal pain processing. J Neural Transm (Vienna). 2019. doi:10.1007/s00702-019-2077-x. PMC7148257 31515656

[58] Patel R, Dickenson AH. Modality selective roles of pro-nociceptive spinal 5-HT2A and 5-HT3 receptors in normal and neuropathic states. Neuropharmacology. 2018;143:29–37. doi:10.1016/j.neuropharm.2018.09.028. 30240783 PMC6277848

[59] McCleane GJ, Suzuki R, Dickenson AH. Does a single intravenous injection of the 5HT3 receptor antagonist ondansetron have an analgesic effect in neuropathic pain? A double-blinded, placebo-controlled cross-over study. Anesth Analg. 2003;97(5):1474–78. doi:10.1213/01.ANE.0000085640.69855.51. 14570668

[60] Tuveson B, Leffler AS, Hansson P. Ondansetron, a 5HT3-antagonist, does not alter dynamic mechanical allodynia or spontaneous ongoing pain in peripheral neuropathy. Clin J Pain. 2011;27(4):323–29. doi:10.1097/AJP.0b013e31820215c5. 21178594

[61] Patel R, Goncalves L, Newman R, Jiang FL, Goldby A, Reeve J, Hendrick A, Teall M, Hannah D, Almond S, et al. Novel TRPM8 antagonist attenuates cold hypersensitivity after peripheral nerve injury in rats. J Pharmacol Exp Ther. 2014;349(1):47–55. doi:10.1124/jpet.113.211243. 24472724

[62] Patel R, Qu C, Xie JY, Porreca F, Dickenson AH. Selective deficiencies in descending inhibitory modulation in neuropathic rats: implications for enhancing noradrenergic tone. Pain. 2018;159(9):1887–99. doi:10.1097/j.pain.0000000000001300. 29863529 PMC6095727

[63] Suzuki R, Rahman W, Hunt SP, Dickenson AH. Descending facilitatory control of mechanically evoked responses is enhanced in deep dorsal horn neurones following peripheral nerve injury. Brain Res. 2004;1019(1–2):68–76. doi:10.1016/j.brainres.2004.05.108. 15306240

[64] Hirsch SJ, Dickenson AH. Morphine sensitivity of spinal neurons in the chronic constriction injury neuropathic rat pain model. Neurosci Lett. 2014;562:97–101. doi:10.1016/j.neulet.2013.10.007. 24128881

[65] Bee LA, Bannister K, Rahman W, Dickenson AH. Mu-opioid and noradrenergic alpha(2)-adrenoceptor contributions to the effects of tapentadol on spinal electrophysiological measures of nociception in nerve-injured rats. Pain. 2011;152(1):131–39. doi:10.1016/j.pain.2010.10.004. 21187272

[66] Little JW, Ford A, Symons-Liguori AM, Chen Z, Janes K, Doyle T, Xie J, Luongo L, Tosh DK, Maione S, et al. Endogenous adenosine A3 receptor activation selectively alleviates persistent pain states. Brain. 2015;138(Pt 1):28–35. doi:10.1093/brain/awu330. 25414036 PMC4285194

[67] Suzuki R, Dickenson AH. Differential pharmacological modulation of the spontaneous stimulus-independent activity in the rat spinal cord following peripheral nerve injury. Exp Neurol. 2006;198(1):72–80. doi:10.1016/j.expneurol.2005.10.032. 16336968

[68] Suzuki R, Matthews EA, Dickenson AH. Comparison of the effects of MK-801, ketamine and memantine on responses of spinal dorsal horn neurones in a rat model of mononeuropathy. Pain. 2001;91(1–2):101–09. doi:10.1016/S0304-3959(00)00423-1. 11240082

